# Developing a tool to assess mattress satisfaction: the Boston Mattress Satisfaction Questionnaire

**DOI:** 10.3389/frsle.2025.1509420

**Published:** 2025-03-12

**Authors:** Rebecca Robbins, Matthew D. Weaver, Laura K. Barger, Stuart F. Quan, Charles A. Czeisler

**Affiliations:** Division of Sleep and Circadian Disorders and Division of Sleep Medicine, Brigham and Women's Hospital and Harvard Medical School, Boston, MA, United States

**Keywords:** mattress, mattress satisfaction, sleep, sleep environment, sleep surface

## Abstract

**Study objectives:**

Adults are advised to spend approximately one third of their lives sleeping, yet there is a dearth of scientific research on mattresses, a common sleep surface. We develop and conduct initial validation of the Boston Mattress Satisfaction Questionnaire (BMSQ).

**Methods:**

The BMSQ was designed with sleep scientists and clinicians (*n* = 5) and mattress industry professionals (*n* = 2) to assess two broad domains: mattress satisfaction (MS) and mattress characteristics (MC), including mattress type, size, age, and extent to which the mattress is pain-inducing. MS is measured with questions assessing mattress comfort, firmness, temperature, and overall satisfaction on 10-point scales from 1 (least) to 10 (most satisfied). We administered the BMSQ to a large, population-based sample of US adults. We also asked demographic questions. We conducted exploratory factor analysis, then dichotomized BMSQ responses (low: ≤ 5; high ≥6) for multivariable logistic regression to explore the demographic characteristics associated with mattress satisfaction.

**Results:**

Among participants (*n* = 1,055), 47.7% were male and 52.2% female. Average age was 49.4 (s.d. = 17.5 years). The 4 BSMQ items demonstrated high inter-item correlation (≥0.8) and Cronbach's α of 0.95. BMSQ-MS variables were inversely correlated with perceptions of the mattress being pain-inducing (*p* < 0.001) and mattress (*p* < 0.001). BMSQ variables had a weak correlation with mattress size (*p* < 0.01). Regression revealed higher mattress satisfaction among those ≥75 years old (v. 18–24 years); Hispanic and Asian individuals (compared to White, non-Hispanic); those earning >$20,000 annually (compared to <$10,000); and those reporting foam, hybrid, air-filled chamber mattresses (compared to all-spring).

**Conclusions:**

Our findings suggest that the BMSQ may be useful for assessing mattress satisfaction.

## 1 Introduction

Adults are advised to spend 7 to 9 h sleeping each day (Hirshkowitz et al., 2015; Watson et al., 2015), yet only about 1 in 3 adults in the U.S. report regularly meeting this recommendation [Centers for Disease Control Prevention (CDC), 2008], and only 3 in 10 adults report their sleep is restorative (Robbins et al., 2022). There are consequences of non-compliance with sleep health recommendations. Insufficient or poor quality or sleep is associated with adverse outcomes, ranging from disturbed mood (Becker et al., 2017; Scott et al., 2006; Wolkow et al., 2019) to cardiovascular disease (Buxton and Marcelli, 2010; Kwok et al., 2018) and cognitive decline (Robbins et al., 2021; Shi et al., 2018). Both intra-individual and environmental factors influence sleep behavior (Bandura, 1969, 1978). Research has explored intra-individual factors, such as psychological beliefs individuals hold about sleep, and their relationship to sleep behaviors (Grandner et al., 2013, 2014; Robbins et al., 2019), and a growing body of literature has examined the broader environment that might influence sleep, such as noise or pollution in the nearby area (Troynikov et al., 2018). A far smaller body of evidence (Bader and Engdal, 2000; Bjorvatn et al., 2018; Jacobson et al., 2008; Radwan et al., 2015; Scharf et al., 1997; Tonetti et al., 2011), however, has explored environmental factors that are the most proximal to the individual, such as the mattress.

Sleeping on a comfortable mattress is frequently a tenet of sleep hygiene recommendations (American Academy of Sleep Medicine D, 2020). Despite this common recommendation, there has been surprisingly little research on the components of mattress satisfaction in the general population. The available literature on mattresses has focused on exploring the mattress types that reduce pain, finding that medium-firm bedding systems, air mattress overlays, and individualized bedding systems are associated with pain reduction (Radwan et al., 2015). Research has also assigned participants to receive either a new foam or new spring mattress and explored sleep over time, finding that outcomes improved in both groups after introducing a new mattress but did not differ by mattress type (Jacobson et al., 2008; Tonetti et al., 2011). In a pilot study, researchers manipulated the softness or firmness of a mattress with a topper, finding no significant differences between sleep outcomes on soft versus firm surfaces (Bader and Engdal, 2000). Another study compared sleep on a traditional spring mattress with that on a foam mattress, finding no difference in sleep outcomes between mattress types (Scharf et al., 1997). Another study assessing the sleep environment (e.g., noise or light in a bedroom), which measured mattress firmness or softness, found that both reports of a firm and soft mattress were associated with more sleep difficulties (Grandner et al., 2022). Bjorvatn and colleagues found that insomnia sufferers were more likely to report lower scores on a Likert scale assessing bed comfort (i.e., “How comfortable is your bed?”). Except for the single item bed comfort question administered in the study conducted by Bjovatn and colleagues, a limitation of the available literature is that firmness designations have been assigned by investigators, without capturing subjective evaluations of mattresses by participants. A further limitation of previous literature is small and convenience sample sizes, which limit our understanding of mattress satisfaction in a manner that is representative of the population.

In 2017, the worldwide mattress industry was valued at 27 billion U.S. dollars (Statista Research Department, 2022), and has been touted as fiercely competitive where mattress manufacturers engage in aggressive advertising campaigns and direct to consumer advertisements. When exploring the available mattress options, consumers are confronted by an array of choices with few rulers or benchmarks with which to evaluate mattress quality or satisfaction. We propose the Boston Mattress Satisfaction Questionnaire (BMSQ) as one such tool for capturing qualitative ratings of mattress satisfaction.

## 2 Materials and methods

### 2.1 Developing the Boston Mattress Satisfaction Questionnaire (BMSQ)

We developed the BMSQ with input from expert scientists, clinicians, and mattress industry professionals in two phases. In the first phase, our expert team, comprised of two physicians and three scientists, discussed the available scientific literature identified through a review of the results from a literature search using the terms: [(mattress OR “sleep surface”) and sleep]. We requested that papers be returned that mentioned those words anywhere in the manuscript. We sorted results (~1,500) using the “Best Match” feature on PubMed.gov and reviewed the first 50 papers that appeared using this search, which included a variety of publication years and papers from a diverse array of fields (i.e., ranging from sleep science to material science). From within these papers, we searched for questions or scales that assessed mattress satisfaction. We extracted the question wording from any studies that collected data from participants having to do with mattress satisfaction and reviewed the question wording. In addition, we interviewed mattress professionals in product development and testing job roles. We asked mattress professionals about components of mattress satisfaction that are commonly reported in their user testing from actual consumers. Finally, we asked our clinical co-authors for their perspective from patients in the clinic regarding mattress satisfaction or issues that may hinder satisfaction that they had uncovered through their years of experience. In the second meeting, the first author developed draft questions that were then discussed and critiqued by the experts. Next, we obtained input on the questionnaire from 2 mattress industry professionals in a focus group. The resulting questionnaire was shared and approved by both groups.

The BMSQ questions that emerged from these formative phases assess two broad domains: mattress satisfaction (MS) and mattress characteristics (MC). The MS questions assess 4 domains of mattress satisfaction: (1) comfort, (2) firmness, (3) temperature, and (4) overall satisfaction. The four satisfaction domains are assessed on 10-point scales from 1 (least satisfied) to 5 (neither satisfied nor unsatisfied) and 10 (most satisfied). To assess these domains, participants are asked “Using a scale of 1 to 10 where 1 is the least satisfied and 10 is the most satisfied, please rate your satisfaction with your current mattress on the following attributes…” with responses that included “...comfort,” “…firmness,” “…temperature,” and “…overall satisfaction.” The four satisfaction items represent the core BMSQ questions.

The MC questions assess the extent to which a person perceives their mattress to be pain-inducing on a scale from 1 (never), 2 (mild pain), 3 (moderate pain), and 4 (severe pain). Participants were also asked several additional questions relating to the age of their mattress (“Approximately how old is your mattress?”), size of their mattress (“What size is your current mattress?”), type of mattress (“What type of mattress do you currently sleep on?”). Mattress type responses were collected on a scale with options that included: (1) springs (composed primarily of metal coils or springs, including “Innerspring” or “Pocket spring”); (2) all foam (composed of polyurethane, latex, “memory foam” or “other foam”); (3) hybrid (composed of springs with a foam or pillow top); (4) water bed; (5) A mattress composed of adjustable air-filled chambers (this is not referring to an inflatable mattress); or an open-ended option: (6) Other. The open-ended responses to the “other” category were organized into the following categories: “futon,” “inflatable air mattress,” “furniture (e.g., couch, chair),” “N/A (do not use a mattress),” and “do not know/unsure.” Finally, participants were asked to report if they share their bed with a bed partner. The full questionnaire can be found in the Supplementary material.

### 2.2 Administering the BMSQ to a population-based sample of adults in the US

Next, we evaluated the BMSQ in a large, population-based sample representative of adults in the U.S., *AmeriSpeak*. *AmeriSpeak* is a probability-based panel managed by the National Opinion Research Center (NORC) at the University of Chicago that provides coverage of more than 97% of the US household population. *AmeriSpeak* is comprised of randomly selected U.S. households sampled by US mail, telephone, and field interview (face to face) using area probability and address-based sampling. Participants in AmeriSpeak are invited to join subsequent panels by web or telephone, then welcomed into any given panel in a stratified sample to assure representativeness with respect to age, gender, race/ethnicity, and education. Additional details describing the AmeriSpeak recruitment and sampling procedures are available in the panel technical report (NORC at the University of Chicago, 2024).

To ensure representativeness of the AmeriSpeak sample in the present analysis, our team compared the resultant AmeriSpeak sample to data from the US Census Bureau (data.census.gov, see the Supplementary material. for statistics from the US Census Bureau: data.census.gov). We qualitatively compare the proportions of age, gender, education, and race/ethnicity between the sample recruited for this study and that of the US Census. We do not perform statistics to compare the percentages, due to the vastly different counts of participants in our study as compared to the US population.

Eligible participants included adults (18 years of age or older) residing in a US household. The current questionnaire was sent to 5,259 participants selected from the AmeriSpeak panel. The survey was administered in September 2021 and took approximately 10 min for participants to complete. Twenty percent (1,055) of participants completed the survey.

### 2.3 Assessing readability of the BMSQ

Finally, we assessed readability of the questionnaire using the Flesch Reading Ease (FRE) and Flesch-Kincaid (F-K) reading formulas (Flesch, 1948; Kincaid et al., 1975). The FRE is a score from 0 to 100 with higher scores indicating greater reading ease, and lower scores indicating more difficult to read. FRE scores 60 and higher indicate readability at the 15-year-old age level. The F-K reading level corresponds to grade level in the United States (US, 1 through 12), with lower levels indicated more accessibility and readability (Flesch, 1948; Kincaid et al., 1975).

### 2.4 Statistical analysis

We employed surveyed weights using the svy command in Stata statistical software (Version 16; StataCorp, College Station, TX) to account for the multistage survey sampling strategy. Descriptive statistics were computed for each of the BMSQ items and plotted to determine the frequency distribution of responses. We explored the internal consistency of the BMSQ using inter-item correlations and Cronbach's alpha. Demographic characteristics of the sample were computed by BMSQ question (comfort, firmness, temperature, and overall satisfaction) and compared using ANOVA with Bonferroni tests. BMSQ responses violated assumptions for Pearson correlation. We confirmed assumptions for Spearman correlation, then computed Spearman rank order correlation coefficients for BMSQ satisfaction measures and questions assessing the extent to which the mattress is pain-inducing, age of the mattress, and size of the mattress. Finally, we performed multivariate regression to identify the demographic and mattress-specific (e.g., type, bed partner) predictors of satisfaction responses. In the regression analyses, we dichotomized mattress satisfaction to either low (a response of “5” or less, which on the satisfaction scale corresponded to neither satisfied nor unsatisfied) or high (a response of “6” or higher), indicating satisfaction. Two-sided hypothesis tests were used with *p* < 0.05 considered to be the threshold for statistical significance.

## 3 Results

Demographic characteristics of the sample (*n* = 1,055) indicate that 51.7% were female, 46% were ≤ 44 years old and 22% were >64 years old. With respect to the highest level of education, 28.7% had a high school diploma and 20.8% had a bachelor's degree or equivalent. Approximately half the sample was married (48.7%). The most commonly reported household income bracket was $50,000–$74,999 (20.1%). Full demographic characteristics are shown in Table 1. The demographic characteristics of the sample are similar to that of the US adult population with respect to age, gender, education, and race/ethnicity (see Supplementary Table S1, for demographic characteristics derived from the US Census in the same year as this study).

**Table 1:** Descriptive statistics summarizing the demographic and Boston Mattress Satisfaction Questionnaire (BMSQ) responses.

	***N***	**Percent %**
**Gender**		
Male	510	48.3%
Female	545	51.7%
**Age**		
18–24	119	11.3%
25–34	185	17.5%
35–44	184	17.5%
45–54	152	14.4%
55–64	182	17.2%
65–74	151	14.3%
75+	82	7.8%
**Education**		
Less than HS	95	9.0%
HS graduate	303	28.7%
Some college	286	27.1%
Bachelor's degree	220	20.8%
Graduate degree	152	14.4%
**Race/Ethnicity**		
White, non-Hispanic	661	62.7%
Black, non-Hispanic	126	11.9%
Other, non-Hispanic	6	0.6%
Hispanic	176	16.7%
2+, non-Hispanic	23	2.1%
Asian, non-Hispanic	64	6.1%
**Marital Status**		
Married	514	48.7%
Widowed	35	3.3%
Divorced	107	10.2%
Separated	52	4.9%
Never married	288	27.3%
Living with partner	59	5.6%
**Income**		
<$10,000	54	5.1%
$10,000–$19,999	107	10.1%
$20,000–$29,999	136	12.9%
$30,000–$39,999	102	9.6%
$40,000–$49,999	65	6.2%
$50,000–$74,999	212	20.1%
$75,000–$99,999	157	14.8%
$100,000–$149,999	134	12.7%
>150,000	89	8.5%
**Pain**		
No pain	744	71.3%
Yes, slight pain	190	18.2%
Yes, moderate pain	84	8.0%
Yes, severe pain	25	2.4%
**Mattress age**		
0–3 years	417	39.8%
4–5 years	264	25.2%
6–7 years	127	12.1%
8–9 years	62	5.9%
10 or more years	178	17.0%
**Mattress size**		
Twin (Twin or XL Twin)	73	7.0%
Full	117	11.1%
Queen	531	50.4%
King (King or California King)	325	30.9%
Other	7	0.6%
**Bedpartner**		
Yes	609	58.2%
No	437	41.8%
**Mattress type**		
Springs	362	34.9%
All foam	311	29.9%
Hybrid	294	28.4%
Adjustable air-filled chambers	51	5.0%
Other/Do not know or unsure	20	1.9%
Do not know/unsure	12	1.1%
Do not use a mattress	2	0.2%
Futon	2	0.2%
Inflatable air mattress	2	0.2%
Furniture (e.g., couch or chair)	1	0.1%
Waterbed	1	0.1%

Descriptive statistics for the remaining items on the BMSQ, indicate that most participants (71.3%) do not experience pain due to their mattress upon waking, but 18.2% report slight pain due to their mattress upon waking, 8.0% report moderate pain due to their mattress, and 2.4% report severe pain due to their mattress. A small majority of participants report sleeping on a queen mattress (50.4%), followed by a smaller number sleeping on king or California king (30.9%). Mattresses most commonly were owned for 0–3 years (39.8%) with 4–5 years as the next most common length of ownership (25.2%). Most respondents (58.2%) reported sleeping with a bedpartner. There was heterogeneity with respect to mattress type with 34.9% sleeping on a spring mattress, 29.9% sleeping on a foam mattress, and 28.4% sleeping on a hybrid mattress. Full descriptive statistics for demographic characteristics of the sample and responses to mattress questions are shown in Table 1.

**Figure 1:**
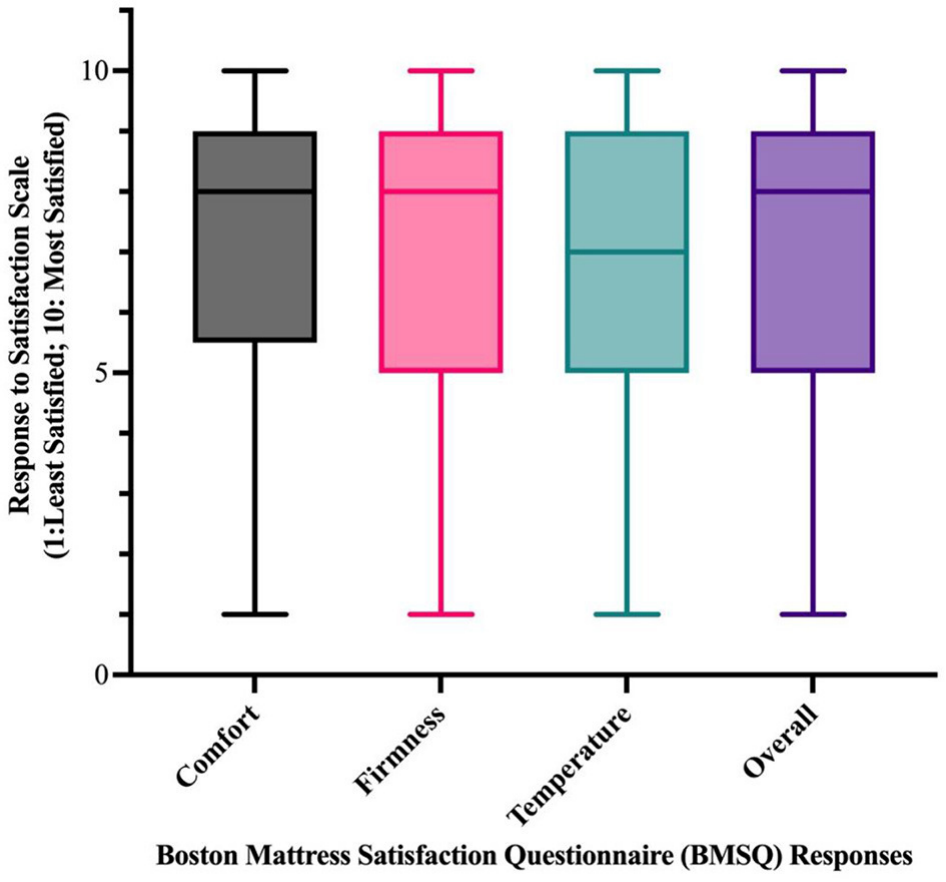
Box and whisker plots describing responses to the four BMSQ satisfaction questions (comfort, firmness, temperature, and overall satisfaction) on the 10-point Satisfaction Scale (1: least satisfied to 10: most satisfied). The box extends from the 25th to the 75th percentile. The line in the middle of the box is plotted at the median.

BMSQ satisfaction responses indicated a mean of 7.1 (95% CI: 6.9–7.27) for comfort, 7.0 (95% CI: 6.8–7.2) for firmness, 6.7 (95% CI: 6.5–6.9) for temperature and 6.9 for overall satisfaction (95% CI: 6.7–7.2). The distribution of responses to the four BMSQ satisfaction domains can be seen in Figure 1. For the core BMSQ satisfaction responses (comfort, firmness, temperature, and overall). The BMSQ MS items demonstrated high internal consistency (Cronbach's α = 0.95).

**Figure 2:**
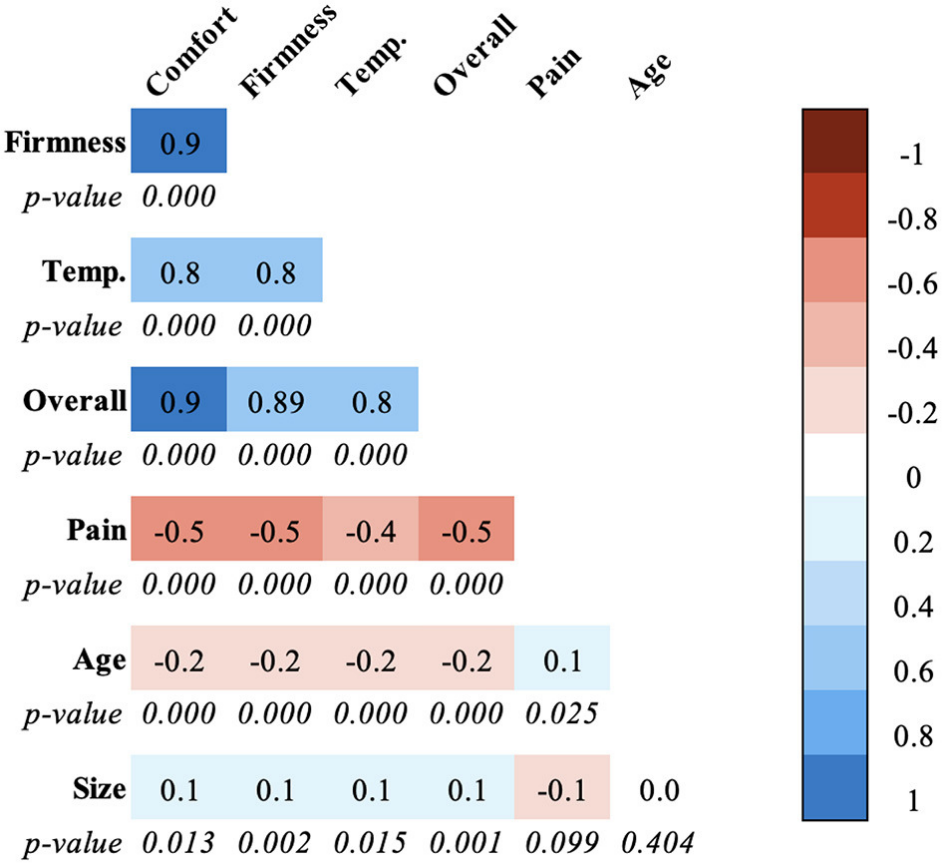
Spearman correlations between Boston Mattress Satisfaction (BMSQ) items relating to mattress satisfaction (comfort, firmness, temperature, overall satisfaction), pain due to the mattress, age of the mattress, and size of the mattress.

The inter-item correlations for BMSQ 4 core satisfaction items were all significant and highly positive (range: *r* = 0.8 to 0.9, *p* < 0.001), as shown in Figure 2. The BMSQ satisfaction items were inversely correlated with pain attributed to the mattress (range: *r* = −0.4 to −0.5, *p* < 0.001). The BMSQ items had a weak, inverse correlation with age of the mattress (all *r* = −0.2, *p* < 0.001). The BMSQ items had a weak correlation with the size of the mattress (all *r* = 0.1, *p* < 0.05), as shown in Figure 2.

A principal components exploratory factor analysis revealed a single factor solution (1 eigenvalue > 1.0) was revealed, with an eigenvalue of 3.2 representing 87.4% of the variance, with a chi-square test = 4646.4, *p* < 0.001. Factor loadings were as follows: comfort (0.95), firmness (0.95), temperature (0.87), and overall satisfaction (0.96).

As shown in Table 2, analyses exploring high/low mattress satisfaction by other mattress characteristics revealed that satisfaction varied by reports of waking with pain due to their mattress (*p* < 0.001) with *post hoc* testing demonstrating that satisfaction was significantly lower among those reporting any level of pain due to their mattress compared to those not reporting pain attributed to the mattress (*p* < 0.001). High/low satisfaction responses also varied by mattress age (*p* < 0.001). Compared to those reporting a mattress in the past 3 years, those reporting a mattress 10 or more years old reported significantly lower satisfaction on all domains (*p* < 0.001). High/low satisfaction also varied by mattress size (*p* < 0.01) for all domains but temperature. *Post hoc* tests reveal that satisfaction is higher among those with a queen (*p* < 0.001) or king mattress (*p* < 0.001) compared to a twin for all domains of satisfaction but temperature. Sleeping without a bed partner reported was associated with lower mattress satisfaction on all domains (*p* < 0.05). Finally, high/low satisfaction varied by mattress type (*p* < 0.001). *Post hoc* test revealed that those sleeping on foam, hybrid, and air-filled chamber mattresses reported higher levels of satisfaction for all domains (*p* < 0.001) compared to those sleeping on all-spring mattresses.

**Table 2:** Exploring differences in high and low mattress satisfaction responses by pain due to the mattress, age of the mattress, size of the mattress, presence of a bed partner, and mattress type.

	**Mattress comfort**					**Mattress firmness**					**Mattress temperature**					**Overall**				
		**95% CI**			***P-* value**		**95% CI**			***P-* value**		**95% CI**			***P-* value**		**95% CI**			***P-* value**
	**Mean**	**Lower**	**Upper**	**F**		**Mean**	**Lower**	**Upper**	**F**		**Mean**	**Lower**	**Upper**	**F**		**Mean**	**Lower**	**Upper**	**F**	
**Pain**				131.8	<0.001				134.1	<0.001				99.9	<0.001				167.8	<0.001
No pain	7.9	6.0	9.8			7.8	5.9	9.8			7.4	5.3	9.6			7.9	5.9	9.9		
Yes, slight pain	5.2	2.7	7.7			5.4	2.9	7.8			5.4	3.0	7.7			5.2	2.7	7.8		
Yes, moderate pain	5.3	2.3	8.3			4.4	1.6	7.1			4.2	1.8	6.7			4.0	1.3	6.7		
Yes, severe pain	3.0	0.2	5.8			3.7	1.0	6.5			3.6	1.6	5.6			2.8	0.1	5.4		
**Mattress age**				12.6	<0.001				14.3	<0.001				12.1	<0.001				14.1	<0.001
0–3 years	7.7	5.4	10.0			7.7	5.3	10.0			7.3	4.9	9.7			7.6	5.2	10.0		
4–5 years	6.8	4.3	9.4			6.8	4.3	9.3			6.6	4.3	9.4			6.8	4.3	9.4		
6–7 years	6.3	3.7	8.9			6.1	3.7	8.9			6.0	3.8	8.9			6.2	3.6	9.0		
8–9 years	6.9	4.2	9.7			6.9	4.3	9.6			6.6	4.1	9.8			6.9	4.3	9.6		
10 or more years	6.5	3.9	9.1			6.5	3.8	9.2			6.0	4.0	9.0			6.2	3.6	9.4		
**Mattress size**																				
Twin or XL twin	5.9	3.3	8.4	4.52	0.001	5.8	3.3	8.4	4.6	0.001	6.0	3.5	8.4	1.84	0.119	5.6	3.0	8.3	6.2	<0.001
Full	7.1	4.7	9.6			7.0	4.6	9.6			6.7	4.6	9.7			6.8	4.4	9.8		
Queen	7.1	4.5	9.7			7.0	4.5	9.7			6.7	4.5	9.7			6.9	4.4	9.8		
King/Cal. King	7.3	4.8	9.7			7.2	4.8	9.7			6.8	4.8	9.7			7.3	4.7	9.8		
**Bedpartner**																				
Yes	7.2	4.8	9.6	4.98	0.026	7.2	4.8	9.6	11.6	0.001	6.8	4.5	9.2	5.65	0.018	7.2	4.7	9.7	10.0	0.002
No	6.9	4.2	9.5			6.7	3.9	9.4			6.5	3.7	9.2			6.6	3.8	9.5		
**Mattress type**																				
Springs	6.0	3.3	8.8	25.8	<0.001	6.0	3.3	8.7	23.36	<0.001	5.9	3.3	8.5	13	<0.001	5.9	3.0	8.7	22.35	<0.001
All foam	7.7	5.6	9.8			7.7	5.6	9.8			7.2	4.9	9.6			7.6	5.4	9.8		
Hybrid	7.4	5.1	9.8			7.3	4.8	9.8			7.0	4.6	9.5			7.4	5.0	9.8		
Air-filled chambers	8.8	7.4	10.3			8.5	6.7	10.2			7.6	5.4	9.7			8.2	5.9	10.5		
Do not know/unsure	7.9	5.4	10.4			8.4	5.7	11.0			6.5	3.1	9.9			7.1	3.4	10.8		
Other	5.6	3.4	7.9			6.3	4.3	8.3			6.4	4.2	8.6			6.2	4.0	8.5		

As shown in Table 3, multivariate regression exploring high/low mattress satisfaction by demographic characteristics indicated that those age 75 and above reported higher satisfaction in each domain compared to those age 18–24 years old. Compared to non-Hispanic White individuals, Hispanic and Asian individuals both reported higher satisfaction in each domain (*p* < 0.05). Finally, compared to the lowest level of income (those earning < $10,000), all other income levels reported higher satisfaction (*p* < 0.05). Compared to those reporting all-spring mattresses, those reporting all foam, hybrid, and air-filled chambers reported higher mattress satisfaction (< 0.001). Compared to those without a bed partner, sleeping with a bed partner was associated with higher overall mattress satisfaction (*p* < 0.05).

**Table 3:** Multivariable regression examining the relationships between high and low mattress satisfaction responses and demographic characteristics.

		**Mattress comfort**				**Mattress firmness**				**Mattress temperature**				**Overall**			
		**B**	**95% CI**		***P-* value**	**B**	**95% CI**		***P-* value**	**B**	**95% CI**		***P-* value**	**B**	**95% CI**		***P-* value**
			**Lower**	**Upper**			**Lower**	**Upper**			**Lower**	**Upper**			**Lower**	**Upper**	
Gender	Male	Reference															
	Female	1.1	0.8	1.5	0.705	1.1	0.7	1.7	0.640	1.3	1.0	1.6	0.056	0.9	0.7	1.3	0.696
Age	18–24	Reference															
	25–34	**0.6**	**0.5**	**0.8**	**< 0.001**	0.8	0.5	1.2	0.268	1.3	1.0	1.7	0.094	0.9	0.3	2.4	0.816
	35–44	0.6	0.2	1.3	0.158	0.8	0.4	1.5	0.448	0.9	0.4	1.8	0.736	1.1	0.4	3.1	0.906
	45–54	0.8	0.3	2.3	0.644	1.5	0.5	4.3	0.426	**2.1**	**1.1**	**4.2**	**0.026**	1.6	0.4	5.6	0.498
	55–64	1.4	0.5	4.0	0.540	**2.2**	**1.2**	**4.0**	**0.015**	**2.6**	**1.5**	**4.6**	**0.001**	2.8	0.8	9.3	0.093
	65–74	1.9	0.5	7.3	0.372	2.2	0.9	5.6	0.098	**5.7**	**2.9**	**11.3**	**< 0.001**	3.7	0.8	16.2	0.088
	75+	**5.9**	**1.8**	**19.2**	**0.004**	**7.6**	**3.2**	**18.0**	**< 0.001**	**10.8**	**4.0**	**29.6**	**< 0.001**	**8.4**	**2.5**	**28.2**	**0.001**
Education	Less than HS	Reference															
	HS graduate	0.8	0.3	2.4	0.710	1.1	0.3	3.5	0.919	0.7	0.5	1.1	0.117	0.6	0.2	1.3	0.159
	Some college	1.0	0.3	3.7	0.942	1.7	0.4	6.4	0.453	1.0	0.4	2.4	0.924	1.0	0.3	3.4	0.948
	Bachelor's degree	1.7	0.8	3.5	0.161	1.8	0.9	4.0	0.115	1.3	0.6	2.8	0.516	1.6	0.9	2.6	0.095
	Graduate degree	1.1	0.3	4.0	0.850	2.2	0.9	5.5	0.099	1.1	0.6	1.8	0.825	1.1	0.5	2.6	0.780
Race/Ethnicity	White, non-Hispanic	Reference															
	Black, non-Hispanic	0.9	0.6	1.3	0.444	0.8	0.6	1.0	0.082	1.0	0.7	1.3	0.792	**0.6**	**0.4**	**0.8**	**0.002**
	Other, non-Hispanic	**3.2**	**1.1**	**9.0**	**0.027**	1.8	0.4	8.8	0.444	0.6	0.3	1.5	0.316	0.5	0.2	1.1	0.085
	Hispanic	**1.3**	**1.0**	**1.6**	**0.024**	**1.9**	**1.5**	**2.3**	**< 0.001**	**1.8**	**1.4**	**2.3**	**< 0.001**	1.3	1.0	1.6	0.058
	2+, non-Hispanic	**0.4**	**0.2**	**0.8**	**0.016**	1.0	0.7	1.4	0.906	1.0	0.4	2.5	0.947	0.6	0.2	1.6	0.312
	Asian, non-Hispanic	**3.1**	**2.1**	**4.6**	**< 0.001**	**3.7**	**2.4**	**5.9**	**< 0.001**	**2.5**	**1.6**	**4.1**	**< 0.001**	**4.0**	**1.0**	**16.0**	**0.049**
Marital status	Married	Reference															
	Widowed	1.6	0.3	8.9	0.570	1.3	0.2	10.4	0.791	1.2	0.7	2.2	0.526	2.5	0.6	11.4	0.228
	Divorced	0.7	0.5	1.0	0.076	0.7	0.5	1.1	0.156	1.0	0.8	1.4	0.854	1.3	0.7	2.3	0.447
	Separated	1.6	0.9	3.0	0.129	0.7	0.3	1.7	0.433	**2.7**	**1.2**	**6.2**	**0.020**	1.9	0.9	4.0	0.074
	Never married	1.6	0.7	3.4	0.246	0.9	0.7	1.1	0.405	**1.4**	**1.1**	**1.8**	**0.005**	**2.0**	**1.2**	**3.6**	**0.013**
	Living with partner	**1.5**	**1.2**	**1.9**	**< 0.001**	0.7	0.4	1.2	0.201	0.9	0.6	1.4	0.592	1.1	0.6	2.0	0.789
Income	<$10,000	Reference															
	$10,000–$19,999	**1.5**	**0.9**	**2.6**	0.091	1.1	0.7	1.7	0.644	1.1	0.5	2.7	0.778	1.1	0.7	1.8	0.698
	$20,000–$29,999	**7.0**	**5.4**	**9.1**	**< 0.001**	1.8	0.8	4.4	0.168	**2.5**	**1.9**	**3.3**	**< 0.001**	**3.8**	**1.9**	**7.6**	**< 0.001**
	$30,000–$39,999	**2.4**	**1.7**	**3.4**	**< 0.001**	1.3	0.7	2.5	0.379	**1.8**	**1.3**	**2.5**	**< 0.001**	1.4	0.5	4.2	0.495
	$40,000–$49,999	**3.3**	**2.5**	**4.5**	**< 0.001**	1.6	0.8	3.1	0.179	**4.6**	**2.8**	**7.7**	**< 0.001**	2.0	0.8	5.0	0.120
	$50,000–$74,999	**4.0**	**2.8**	**5.7**	**< 0.001**	**1.6**	**1.1**	**2.4**	**0.027**	**2.6**	**1.9**	**3.6**	**< 0.001**	**2.8**	**1.3**	**6.2**	**0.010**
	$75,000–$99,999	**2.9**	**2.1**	**4.1**	**< 0.001**	1.4	0.9	2.1	0.101	**3.9**	**2.2**	**6.8**	**< 0.001**	**2.2**	**1.1**	**4.4**	**0.022**
	$100,000–$149,999	**7.2**	**3.9**	**13.0**	**< 0.001**	**2.8**	**1.6**	**4.9**	**< 0.001**	**4.5**	**2.5**	**8.2**	**< 0.001**	**4.1**	**2.6**	**6.6**	**< 0.001**
	>$150,000	**7.6**	**5.2**	**11.0**	**< 0.001**	**2.8**	**1.9**	**4.3**	**< 0.001**	**5.5**	**3.4**	**8.8**	**< 0.001**	**4.5**	**2.4**	**8.5**	**< 0.001**
Mattress	All springs	Reference															
Type	All foam	**3.5**	**3.0**	**4.1**	**< 0.001**	**3.7**	**2.8**	**4.9**	**< 0.001**	**3.3**	**2.0**	**5.2**	**< 0.001**	**3.5**	**3.0**	**4.2**	**< 0.001**
	Hybrid	**3.0**	**2.3**	**4.0**	**< 0.001**	**2.3**	**1.5**	**3.4**	**< 0.001**	**2.6**	**2.0**	**3.4**	**< 0.001**	**3.5**	**2.6**	**4.6**	**< 0.001**
	Air filled chambers	**20.5**	**5.8**	**73.2**	**< 0.001**	**5.7**	**4.3**	**7.4**	**< 0.001**	**3.4**	**2.0**	**5.6**	**< 0.001**	**5.2**	**3.8**	**7.1**	**< 0.001**
	Other	**0.6**	**0.4**	**0.9**	**0.019**	1.1	0.7	2.0	0.624	0.8	0.4	1.5	0.469	0.9	0.5	1.8	0.822
Bed partner	Yes	Reference															
	No	1.1	0.6	2.2	0.718	1.2	0.9	1.5	0.295	1.1	0.8	1.7	0.501	**1.7**	**1.0**	**2.7**	**0.039**

Bold indicates significance *p* < 0.05.

With respect to the readability and accessibility of the scale, the BMSQ scored a 68 on the FRE. In addition, the BMSQ scored at a 6th grade reading level on the F-K.

## 4 Discussion

We designed and conducted the initial validation of the BMSQ. The BMSQ assesses two broad domains, including Mattress Satisfaction (MS) and Mattress Characteristics (MC). There are 4 core questions assessing mattress satisfaction, including satisfaction with mattress comfort, firmness, temperature, and overall satisfaction. While researchers have rigorously developed tools to assess the overall sleep environment (i.e., presence of light or smell in the bedroom; Grandner et al., 2022), no scale exists that is focused solely on mattress satisfaction and characteristics. The first part of the BMSQ, designed to assess Mattress Satisfaction, could be delivered alone, or together with the second part, Mattress Characteristics. The questions assessing Mattress Characteristics likely will be most appropriate for researchers who might be interested in examining mattress satisfaction by mattress characteristics (e.g., mattress size or age), while industry professionals (e.g., bed manufacturers or independent testing agencies) may be interested in delivering the mattress satisfaction (Part 1) questions alone. The BMSQ is designed to be flexible and adapt to different needs and applications.

We tested the BMSQ in a large, population-based sample that is representative of adults in the U.S. Our results indicate that these core BMSQ satisfaction questions have high inter-item correlation and reliability. Our study found that the most common length of mattress ownership was 3 or fewer years of ownership, which is in contrast to previous research that found the mean ownership of a mattress was 9.5 years (Jacobson et al., 2008). It is possible that the increasingly competitive nature of the mattress industry and their direct to consumer advertisements (Statista Research Department, 2022) has increased purchases of mattresses across the U.S., shifting the mean years of mattress ownership lower.

Overall, respondents reported a mean mattress satisfaction score of 6.9, which indicates individuals are somewhat satisfied with their current mattress. Our results demonstrate that the BMSQ mattress satisfaction questions vary by demographic factors, with higher levels of satisfaction among older individuals (age 75 and above), both Hispanic and Asian individuals, and individuals with higher income levels. It is interesting that, in multivariable regression, our study uncovered higher levels of mattress satisfaction in older adults after controlling for income. This is interesting, because many older adults struggle with sleep (Ohayon and Reynolds, 2009), which could impact their perceptions of mattress satisfaction. Future research is needed to combine measures of sleep satisfaction with the proposed measures of mattress satisfaction to fully understand the relationship between sleep and mattress satisfaction.

Our results offer support for the BMSQ core mattress satisfaction questions as internally consistent measures of satisfaction. Moreover, our study found higher levels of mattress satisfaction on the BMSQ was inversely associated with mattress age, indicating that older mattresses offered lower levels of satisfaction and were inversely associated with pain, whereby those experiencing pain due to their mattress also reported lower levels of satisfaction. Additionally, size and type are associated with mattress satisfaction. Sleeping with a queen or king mattress scored higher on satisfaction domains of comfort, firmness, and temperature compared to a twin mattress. Sleeping on an air-filled chamber bed was associated with the greatest comfort, firmness, temperature, and overall satisfaction, whereas sleeping on a spring only mattress was the least satisfying of the alternatives—with hybrid and foam mattresses between the two. We also found that bed partner was associated with overall mattress satisfaction, which is consistent with the literature demonstrating that having a partner, who could also be a bed partner, is a source of social support and financial stability, and associated with better happiness and health (Stack and Eshleman, 1998).

The BMSQ performed satisfactorily on validated metrics of readability and accessibility. Specifically, the BMSQ received a score of 68 on the FRE, which indicates moderate reading ease. The scale scored at a 6th grade reading level, which indicates that the scale is accessible to anyone who has completed grade 6 or higher in the US.

According to a study funded by the Better Sleep Council, the consumer-education arm of the International Sleep Products Association, more than half of participants reported taking between 3 days and 2 weeks to research mattresses before making a decision (Palm, 2016). This suggests that consumers place a large amount of time researching mattress options before making a purchase. There are several resources consumers may consult when making a mattress decision, such as Consumer Reports (2023) or the New York Times (Wirecutter, 2023), yet few of these resources are guided by scientific evidence or employ scientifically vetted assessments in their evaluations. Moreover, there is an adaptation period for mattresses that lasts for several days or longer (Bader and Engdal, 2000), further complicating a consumer's ability to pick an optimal mattress in a store given that it is likely to take time for them to adapt to the surface. The BMSQ has potential to provide such a ruler against which future reviews or recommendations for mattresses are offered to consumers.

It is notable that mattress prices have increased dramatically, and may continue to do so as mattress companies are increasingly considering elements such as sensor technology, into their mattresses (Technavio, 2024). It is possible that consumers who conduct extensive research on a mattress and/or purchase a higher priced mattress may be subject to cognitive biases that interfere with the actual mattress experience, biasing them toward increased satisfaction.

The BMSQ is, to our knowledge, the first questionnaire to explore self-reported mattress satisfaction and assess mattress characteristics. Jacobson assessed ‘sleep comfort' (Jacobson et al., 2008), but not specifically about comfort due to the mattress. Although each of the mattress satisfaction questions are intended to capture important components of the mattress experience, the importance of satisfaction with the temperature of a mattress may be increasingly important in the face of our current climate crisis. According to a study of more than 765,000 participants between 2002 and 2011, rising nighttime temperatures were observed and found to correlate with self-reports of poor sleep quality (Obradovich et al., 2017). Individuals from disadvantaged communities and minoritized populations who do not have access to air conditioning are particularly susceptible to the adverse effects of high temperatures on sleep (Williams et al., 2022). Unfortunately, these challenges relating to rising temperatures and sleep decrements may be exacerbated in the years to come as the climate crisis worsens.

### 4.1 Limitations

It is a limitation that the BMSQ was designed with a small panel of sleep researchers, clinicians, and industry professionals, which were not necessarily representative of all sleep researchers, clinicians, or industry professionals. It is a further limitation that we did not capture either the precise duration of mattress ownership, but instead asked for participants to report the age of their mattress within several ranges of years, which precluded our ability to calculate a mean ownership duration; mattress price, which could bias responders; or weight and height of responders, which could also influence mattress satisfaction. It is also a limitation that our study relied on self-reported measures of mattress satisfaction and that some of the mattress satisfaction questions may qualitatively appear to be similar (e.g., “comfort” and “firmness”). We capture mattress satisfaction at a single point in time in this study. It would be interesting for future research to explore how responses to these questions evolve over time. We realize this may be limiting, as a continuous measure can be advantageous in certain contexts. Also, the clinicians who contributed to the design of the questionnaire indicated that capturing patient-reported pain due to a mattress is an important element to measure, given that it could hinder mattress satisfaction. However, the question capturing pain due to a mattress on the BMSQ (“Do you ever experience pain upon waking due to your mattress?”) could be perceived as leading. Questions regarding bed sharing were limited to yes/no response options and did not include pets. Additional response options could be useful in this area. It is important to note that the BSMQ does not capture sleep satisfaction, which could be important for understanding how a mattress supports sleep itself. It is a limitation that consumers were not engaged, such as in focus groups, during the scale development to ensure the face validity of the BMSQ. The present analysis did not afford the ability to conduct convergent, discriminant, or criterion validity, which is a limitation. Applying rigorous analytic techniques such as item response theory approaches, may further clarify and aid in the refinement of the items on the BMSQ. In addition, we did not measure gold standard criterion, such as date of mattress purchase, to ensure self-reported mattress age was accurate. Finally, it is a limitation that the response rate was relatively low while the panel employed in this sample was a large, population-based sample that is nationally representative of adults in the US; it is possible that selection bias limited the generalizability of our findings.

## 5 Conclusions

We develop and conduct initial validation of the BMSQ. We explore the reliability and the internal consistency of the BMSQ for assessing mattress satisfaction and characteristics. Our results indicate the tool is internally consistent and associated with anticipated factors, such as mattress age and size. The BMSQ may be a viable tool for assessing mattress satisfaction among customers, researchers, and industry professionals, but future research is needed to rigorously evaluate and validate the BMSQ against gold standard measures of the bedroom environment and sleep.

## Data Availability

The raw data supporting the conclusions of this article will be made available by the authors, without undue reservation.
