# Supplementary material for: Developing a tool to assess mattress satisfaction: the Boston Mattress Satisfaction Questionnaire
Source: Front Sleep. 2025 Mar 12;4:1509420. doi: 10.3389/frsle.2025.1509420 (PMC12713840; doi:10.3389/frsle.2025.1509420)
Supplement: Supplementary file 1 [file Data_Sheet_1.docx]

**Supplementary Tables**

**Supplement 1**

**Descriptive statistics of US adults according to the US Census Bureau (2022)**

| **Variable** | **Category** | **%** |
| --- | --- | --- |
| Age | 18-29 | 20.5% |
|  | 30-44 | 25.2% |
|  | 45-59 | 24.5% |
|  | 60+ | 29.8% |
| Gender | Male | 48.3% |
|  | Female | 51.7% |
| Education | Less than high school diploma | 9.8% |
|  | High school diploma or equivalent | 28.2% |
|  | Some college/ associates | 27.7% |
|  | Bachelor's degree or above | 34.3% |
| Race/Ethnicity | White, non-Hispanic | 62.8% |
|  | Black, non-Hispanic | 11.90% |
|  | Hispanic | 16.70% |
|  | Asian, non-Hispanic | 6.40% |
|  | Other | 2.20% |

**Supplement 2**

**The Boston Mattress Satisfaction Questionnaire (BMSQ)**

| **Part I: Mattress Satisfaction (MS)** | | | | | | | | | | | |
| --- | --- | --- | --- | --- | --- | --- | --- | --- | --- | --- | --- |
| **MS. Using a scale of 1 to 10 where 1 is the least satisfied and 10 is the most satisfied (a 5 indicates being neither satisfied nor unsatisfied), please rate your satisfaction with your current mattress on the following attributes…** | | | | | | | | | | | |
|  | | **Least Satisfied** |  |  |  |  |  |  |  |  | **Most Satisfied** |
| MS1. The comfort of your mattress: | | **1** | **2** | **3** | **4** | **5** | **6** | **7** | **8** | **9** | **10** |
| MS2. The firmness of your mattress: | | **1** | **2** | **3** | **4** | **5** | **6** | **7** | **8** | **9** | **10** |
| MS3. The temperature of your mattress: | | **1** | **2** | **3** | **4** | **5** | **6** | **7** | **8** | **9** | **10** |
| MS4. Your overall satisfaction with your mattress: | | **1** | **2** | **3** | **4** | **5** | **6** | **7** | **8** | **9** | **10** |
| **Part II: Mattress Characteristics (MC)** | | | | | | | | | | | |
| **Please mark your response to the following questions regarding your current mattress:** | | | | | | | | | | | |
| MC1. Do you ever experience pain upon waking due to your mattress? | 1. No, I do not experience pain upon waking due to my mattress.  2. Yes, I experience slight pain upon waking due to my mattress.  3. Yes, I experience moderate pain upon waking due to my mattress.  4. Yes, I experience severe pain upon waking due to my mattress. | | | | | | | | | | |
| MC2. Approximately how old is your current mattress? | 1. 0-3 years  2. 4-5 years  3. 6-7 years  4. 8-9 years  5. 10 or more years | | | | | | | | | | |
| MC3. What size is your current mattress? | 1. Twin (Twin or XL Twin)  2. Full  3. Queen  4. King (King or California King)  5. Other ______________________ | | | | | | | | | | |
| MC4. Do you share your bed with a partner? | 1. Yes  2. No | | | | | | | | | | |
| MC5. What type of mattress do you currently sleep on? | 1. Springs (composed primarily of metal coils/springs, such as “Innersprings” or “Pocket springs”)  2. All foam (composed of polyurethane, latex, “memory” or other foam)  3. Hybrid (composed of springs with a foam or pillow top)  4. Water bed  5. A mattress composed of adjustable air-filled chambers (this is not referring to an inflatable mattress)  6. Other _____________________ | | | | | | | | | | |

© Division of Sleep and Circadian Disorders, Brigham and Women’s Hospital

***BMSQ Instructions for Scoring***:

Each of the four mattress satisfaction dimensions (MS1-MS4) of the BMSQ are intended to be analyzed individually. For interpretation of each dimension, it is recommended that ratings of <= 5 indicate low satisfaction, while responses of >=6 indicate higher satisfaction.

Each of the five mattress characteristics items (MC1-MC5) do not have scores or rankings associated with the respective response options. Rather, the MC items are intended to provide supporting contextual information about the respondent’s current mattress that may aid interpretation of the scores on the four mattress satisfaction dimensions.
